# Cost of a lymphedema treatment mandate-10 years of experience in the Commonwealth of Virginia

**DOI:** 10.1186/s13561-016-0117-3

**Published:** 2016-09-02

**Authors:** Robert Weiss

**Affiliations:** 10671 Baton Rouge Avenue, Porter Ranch, CA 91326 USA

**Keywords:** Lymphedema treatment costs, Healthcare costs, Health insurance, Medical claims, Insurance mandates, Chronic disease management, Economic analysis, Treatment benefits

## Abstract

Treatment of chronic illness accounts for over 90 % of Medicare spending. Chronic lymphedema places over 3 million Americans at risk of recurrent cellulitis. Health insurers and legislators have taken an active role in fighting attempts to mandate the treatment of lymphedema for fear that provision of the physical therapy and compression materials would result in large and uncontrollable claim costs. The author knows of no open source of lymphedema treatment cost data based on population coverage or claims. Published studies compare cost of treatment versus cost of non-treatment for a select group of lymphedema patients. They do not provide the data necessary for insurance underwriters’ estimations of expected claim costs for a larger general population with a range of severities, or for legislators’ evaluations of the costs of proposed mandates to cover treatment of lymphedema according to current medical standards. These data are of interest to providers, advocates and legislators in Canada, Australia and England as well as the U.S.

The Commonwealth of Virginia has had a lymphedema treatment mandate since 2004. Reported data for 2004–2013, representing 80 % of the Virginia healthcare insurance market, contains claims and utilization data and claims-based estimates of the premium impact of its lymphedema mandate. The average actual annual lymphedema claim cost was $1.59 per individual contract and $3.24 per group contract for the years reported, representing 0.053 and 0.089 % of average total claims. The estimated premium impact ranged 0.00–0.64 % of total average premium for all mandated coverage contracts. In this study actual costs are compared with pre-mandate state mandate commission estimates for proposed lymphedema mandates from Virginia, Massachusetts and California.

Ten years of insurance experience with a lymphedema treatment mandate in Virginia shows that costs of lymphedema treatment are an insignificant part of insured healthcare costs, and that treatment of lymphedema may reduce costs of office visits and hospitalizations due to lymphedema and lymphedema-related cellulitis. Estimates based on more limited data overestimate these costs. Lymphedema treatment is a potent tool for reduction in healthcare costs while improving the quality of care for cancer survivors and others suffering with this chronic progressive condition.

## Background

One of our most urgent societal problems today is our inability to afford quality health care. Treatment of chronic illnesses now accounts for almost 93 % of Medicare spending.^1^ Lymphedema, once acquired, is a lifelong progressing disease with no currently known cure. Chronic lymphedema places over 3 million Americans at risk of recurrent cellulitis. Yet without convincing predictions of the costs and benefits of lymphedema treatment, insurers are reluctant to fully cover treatment of this common condition. The quality of available treatment often does not meet the recommended standards of knowledgeable lymphedema specialty groups such as the International Society of Lymphology (ISL), the American Lymphedema Framework Project (ALFP), the American Cancer Society (ACS) and the National Lymphedema Network (NLN). These standards include an intensive treatment phase by specially qualified therapists, including, as required: manual lymph drainage; multiple layers of short-stretch bandages and padding; range of motion exercises; and patient instruction in self-treatment. A home care maintenance phase includes: the provision and daily use of compression bandages, garments and devices; meticulous skin care; decongestion exercises; and repeated light massage as required [1]. Insurance coverage of these elements of treatment is sporadic, often driven by “pound-foolish” fiscal policies based on fear of unconstrained expense and ignorance of the preventive value of early intervention and effective home management.

Legislation has been introduced in recent years in many states to mandate the treatment of lymphedema according to current medical standards. Only two state legislatures have been successful in enacting lymphedema mandates, ie Virginia in 2003 and North Carolina in 2009. The Virginia Code has provisions for reporting separately the costs of every health mandate, and it is the series of these reports for the first 10 years of operation that is the basis of this review.

## Data sources

### Virginia lymphedema treatment mandate

The Commonwealth of Virginia (COVA, VA or Virginia) was the first state to introduce a lymphedema treatment mandate covering the cost of the treatment of lymphedema from all causes. The lymphedema mandate, House Bill 383, was introduced by Delegate Leo C. Wardrup, Jr. in 2002, and reintroduced and passed in 2003 as Virginia House Bill 1737.^2^

The Virginia lymphedema treatment mandate became effective on January 1, 2004. Defined in section 38.2–3418.14 of the Code of Virginia, it requires insurers, health services plans, and HMOs to provide coverage for the treatment of lymphedema, including benefits for equipment, supplies, complex decongestive therapy, and outpatient self-management training and education.

### Annual data reports

Virginia also has in place a statute^3^ that requires every insurer, health services plan and health maintenance organization (HMO) that underwrites more than $500,000 of accident and sickness insurance subject to the mandate, to segregate and report to the State Corporation Commissioner the yearly cost and utilization information for each of the mandates currently in effect. The Commission is required to prepare a consolidation of these reports for annual submission to the Governor and the General Assembly. This collection of annual reports,^4^ which includes the annual costs of the lymphedema treatment mandate in Virginia, constitutes the most complete, non-proprietary population-based data set known to the author that documents the actual insurance cost of lymphedema treatment.

### Mandate commission reports for California, Massachusetts and Virginia

Lymphedema treatment mandates similar to the one passed by Virginia have been and are being considered in a number of other states. Most states require that proposed health care legislation be analyzed by an independent expert commission to determine its impact on the state before being passed on to the legislature for decision. These mandate commissions analyze the projected medical, societal and financial impacts of the legislation and make recommendations to the legislature based on their studies. Analysis reports usually include a determination of financial impact through analysis of insurance claims databases, which are sparse, proprietary, and incomplete for lymphedema. The special mandate commission reports for proposed lymphedema mandates in California, Massachusetts and Virginia^5^^,^ 6 ^,^ 7 are summarized and compared to the cost data accrued during 10 years of actual operation in Virginia.

### Benefits Not included in mandate reports

Mandate costs reported represent spending impacts only, and do not consider the projected beneficial effects of lymphedema treatment in reducing medical and hospital costs through the inevitable reduction of lymphedema-related cellulitis and disability [2, 3] and reduction of the need to treat the psychosocial effects of lymphedema. From an insurance viewpoint the results are conservative since they define only the costs, and not the resulting benefits, of lymphedema treatment.

## Methods

### Annual reports to the Virginia governor and legislature

The source of data for this study is the series of annual reports of cost and utilization information for health benefits mandated by the Code of Virginia.^3^ Insurers, health services plans, and health maintenance organizations (HMOs) report to the State Corporation Commission, which in turn prepares a consolidated report each year for submission to the Virginia Governor and General Assembly.

Ten years of actual cost and utilization data for the lymphedema mandate were abstracted from the annual reports for the 30 Virginia healthcare mandates (Table 1). This Virginia report series is a non-proprietary, comprehensive and authoritative source of lymphedema treatment cost data. The data covers 17–28 major insurance companies and 10–16 HMOs representing 77–81 % of the Virginia health insurance market, numbering 1.0–1.7 million units of coverage each year (Table 2).

**Table 1:** Data reports used in this study

Private, group, HMO contracts			Pre-mandate contracts		
Data year	Report year	Report no.	Data year	Report year	Report no.
2004	2005	RD191	1999	2001	HD007
2005	2006	RD289	2000	2002	HD010
2006	2007	RD246	2001	2003	HD008
2007	2008	RD322	2002	2003	RD049
2008	2009	RD294	2003	2004	RD110
2009	2010	RD300	State employee contracts		
2010	2011	RD281	2009–10	2011	RD146
2011	2012	RD290	2010–11	2011	RD381
2012	2013	RD300	2011–12	2012	RD379
2013	2014	RD335	2012–13	2013	RD415
2014	2015	N/A	2013–14	2014	RD410

Reports available at Virginia's legislative information system website. http://leg2.state.va.us/DLS/h&sdocs.nsf/Search+All/?SearchView&SearchOrder=4&query=38.2-3419.1 Accessed October 4, 2016

**Table 2:** Population coverage of data sources

Type of insurer	Calendar year										
	2004	2005	2006	2007	2008	2009	2010	2011	2012	2013	Mean
Insurers											
Number	28	27	28	26	26	25	24	17	18	20	23.9
% of Market	47.03	47.42	48.8	50.32	51.6	51.34	53.66	52.79	50.29	50.59	50.384
Coverage Units	729,466	829,595	820,409	973,469	1,008,671	809,954	984,643	718,378	562,198	576,622	801,341
HMOs											
Number	15	16	14	14	14	14	11	10	10	10	12.8
% of Market	33.2	32.92	28.46	29.7	28.36	28.43	27.83	28.59	28.69	27.65	29.383
Coverage Units	640,417	657,841	662,454	745,460	686,321	705,604	698,580	615,280	450,338	526,698	638,899
Totals											
Number	43	43	42	40	40	39	35	27	28	30	36.7
% of Market	80.23	80.34	77.26	80.02	79.96	79.77	81.49	81.38	78.98	78.24	79.767
Coverage Units	1,369,883	1,487,436	1,482,863	1,718,929	1,694,992	1,515,558	1,683,223	1,333,658	1,012,536	1,103,320	1,440,240

Source: Data abstracted from EXECUTIVE SUMMARIES of Private, Group, HMO Contract reports

In addition to the 10 annual mandate reports covering private insurance, group insurance, and HMOs,^8^ separate annual reports cover Virginia State insurance and Medicaid contracts. Costs and utilization summaries for the lymphedema mandate are summarized for the three State-contracted insurers over the 5-year period of 2010–2014 representing an additional 5–6 % of the Virginia insurance market (Table 3).

**Table 3:** Claims data for state health benefit plans

VA Health Benefit Plan	Fiscal year (July-June)				
	2010	2011	2012	2013	2014
Anthem total claims paid					
Visits/Year	438	301	327	438	528
Days/Year	0	28	52	0	33
Claim Cost/Contract	$0.48	$0.39	$0.87	$0.53	$0.88
Administrative cost	$755	$614	$1,210	$934	$1,435
Total claim payments	$39,728	$32,297	$71,196	$44,480	$75,538
Number of contracts	82,533	82,281	82,132	83,618	86,287
Optima total claims paid					
Visits/Year	16	12	15	13	
Claim Cost/Contract	$0.48	$0.71	$1.18	$1.03	
Administrative cost	$703	$1,030	$1,546	$1,124	
Total claim payments	$4,321	$6,329	$9,497	$6,904	
Number of contracts	8,922	8,860	8,074	$6,721	
Aetna total claims paid					
Visits/Year					42
Claim Cost/Contract					$0.27
Administrative cost					$0
Total claim payments					$15,495
Number of contracts					57,984

Source: Data abstracted from State Employee Contracts reports

A series of annual mandate reports for the 5 years preceding the introduction of the lymphedema mandate was examined to determine whether introduction of lymphedema treatment affected healthcare cost to any significant degree (Table 1).

The Virginia lymphedema treatment costs are analyzed for quality and trends, and then compared with projections made in four pre-legislative lymphedema mandate impact analyses.^5,^ ^6,^ ^7,^ 9

### CPT and ICD-9-CM codes collected

The data collection and reporting rules^3,^ 10 ^,^ 11 require insurers to use standard medical procedure and diagnosis codes when developing claim information for each benefit category. Benefit costs have been defined in this manner to ensure a reasonable level of consistency among data collection methodologies employed by the various insurers. The codes utilized in the preparation of these reports are part of two widely accepted coding systems used by most hospitals, health care providers, and companies. These code systems are outlined in the Physicians’ Current Procedural Terminology (CPT-Plus) for medical procedures and the International Classification of Diseases - 9th Revision - Clinical Modification (ICD-9-CM) for medical diagnoses.

The codes collected and priced in the company claims reports include ICD-9-CM lymphedema diagnostic codes: 457.0 Postmastectomy lymphedema syndrome; 457.1 Other lymphedema; and 757.0 Hereditary edema of legs, and lymphedema treatment CPT codes: 97124 Massage, compression; 97140 Manual therapy techniques, manipulation; and 97535 Self-care/home management training.^10^

### Population Coverage

The sources of the data analyzed in this study are summarized in Table 1. Over the 10 years considered in this study, 2004–2013, an average (range) of 23.9 (17–28) insurers and 12.8 (10–16) HMOs provided insurance coverage to approximately 1.44 million Virginians each year (Table 2).

The portion of the insured population in Virginia covered by these reports approached 80 %. Addition of reports for 2010–2014 for State-insured employees and Medicaid subscribers (Table 3) brings the coverage data for 2010–14 to over 85 % of the health insurance policies underwritten in Virginia.

This study updates and expands the data results described in Stout, Weiss, Feldman et al. [4] from 7 to 10 years’ of claims history, presents the raw data and not just the average and range of the first 7 years, and provides a more detailed analysis of the data and data trends. The detailed information on the three mandate reports from California, Massachusetts and Virginia is not herein repeated, as it is available on Table 3 of Reference [4].

### Statistical tools

Microsoft® Excel® 2008 for Mac Version 12.3.6 installed on the author’s Apple iMac under OSX Version 10.9.5 operating system was utilized to process the data and prepare the charts. The mathematical functions AVERAGE, STDEVP AND SLOPE were utilized to determine the means, standard deviations, and slopes of the data, respectively.

## Results

### Claim experience

Claim experience is a direct measure of the cost of lymphedema treatment and is the focus of this study.

Companies reported their claim experience for each mandated benefit for the calendar year. Instructions to companies filling out the input forms^11^ are explicit that the reported total claims used to determine percentage of total claims includes “all claims paid or incurred under the types of policies subject to the reporting requirements… and not the total claims paid or incurred for the mandate.”

**Fig. 1:**
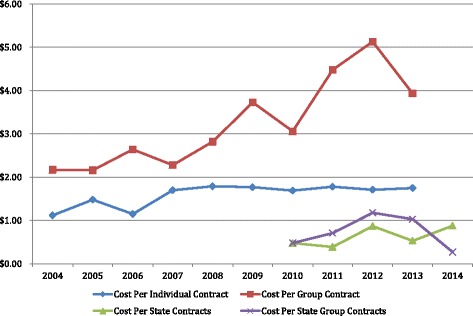
Lymphedema Claim Experience, Individual & Group Policies. Source: Data abstracted from Tables 5 & 6 of Private, Group, HMO Contract reports and from MB-1 Forms in State Employee Contracts reports

**Fig. 2:**
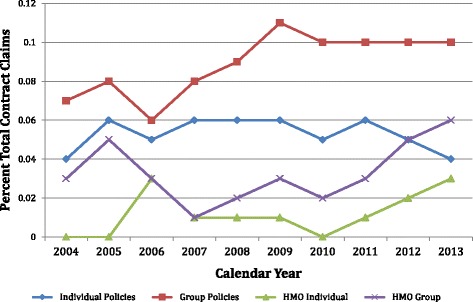
Lymphedema Claims Experience, All Policies, Percent of Total Contract Claims. Source: Data abstracted from Tables 5, 6 and 18 of Private, Group, HMO Contract reports and from MB-1 Forms in State Employee Contracts reports

Tables 5 and 6 of the Virginia report series^4^ summarize the average claim cost per contract or certificate for each mandate, and the average percentage of total contract claims that these costs represent. Claim costs per contract and percent of average contract claims for the lymphedema treatment claims are summarized on Figs. 1 and 2 for 10 years of effectivity of the Virginia lymphedema mandate from 2004 through 2013.

### Claim costs

The average annual lymphedema claim costs per individual contract^8^ was $1.59 (Range $1.12–$1.79), and per group contract^8^ $3.24 ($2.16–$5.13) (Fig. 1). Special reports were issued starting in 2011 (Table 1) with partial reports of claim experience for Virginia State employees and Medicaid patients for reporting periods starting with Fiscal Year 2010 (July 1, 2009 through June 30, 2010) (Fig. 1). The average annual lymphedema costs per contract over 5 years were $0.63 ($0.39–$0.88) for 83,370 individual State contracts, and $0.85 ($0.48–$1.18) for 8,144 group State contracts.

No attempt was made to combine the individual and State data as they were collected over staggered time periods, ie private insurance and HMO data is for the calendar year while State insurance data is for the fiscal year and covered different periods. The claim costs for the State employee and Medicaid insurance are uniformly lower than similar individual and group policies (Fig. 1). Combining the data would distort analysis of 10-year claim cost trends.

### Lymphedema claims as a percentage of total contract claims

The lymphedema claims filed as a percentage of the total contract claims for individual contracts was 0.05 % (Range 0.04–06 %), and for group contracts was 0.09 % (0.06–0.11 %) (Fig. 2). The percentage of lymphedema claims to all HMO contract claims was lower, averaging 0.01 and 0.03 % for individual and group contracts.

### Utilization of benefits

Claim information regarding the rate of utilization of the mandated benefits is also reported. Companies are required to report the number of visits and the number of days of hospitalizations attributable to each mandated benefit for which claims were paid (or incurred) during the reporting period. This analysis focuses exclusively on group business because the group data is believed by the State to be significantly more reliable than that reported for individual business.^4^

**Fig. 3:**
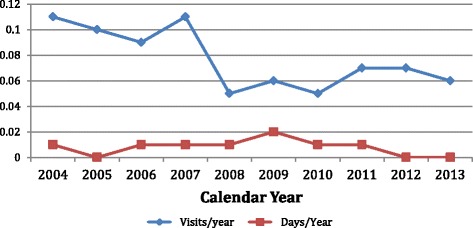
Utilization of Lymphedema Services. Source: Data abstracted from Table 7 of Private, Group, HMO Contract reports

Tables 7 of the Virginia report series^4^ represent utilization of services in terms of the average annual number of visits per certificate for each benefit, and the average number of days per year per certificate for each benefit. These data are collected for the lymphedema mandate and plotted in Fig. 3.

“Number of Visits” refers to the number of provider and physician visits, and “Number of Days” refers to the number of inpatient or partial hospital days. The numbers reported are intended to be consistent with the type of service rendered. For example, “number of days” are not reported unless the claim dollars being reported were paid or incurred for inpatient or partial hospitalization.

The number of visits for lymphedema treatment averaged 0.1 (Range 0.09–0.11) visits per year for the first 4 years of the mandate (Fig. 3), and dropped to 0.06 (0.05–0.07) visits per year for the last 6 years. Hospitalizations for lymphedema remained at or below 0.02 days per year during the entire 10-year period with a downward trend during the last 5 years.

The number of provider visits for lymphedema treatment per year per contract for the three State insurers ranged from 0.0007 to 0.0049 (Table 3). Hospitalizations were not reported by the State insurers.

### Premium impact

Companies are required to use “actual claim experience and other relevant actuarial information” to determine the premium impact of each mandated benefit.^11^ Companies do not specify an additional cost of coverage for a mandated benefit. An exception may occur with mandated offers of coverage. For those companies that do not include the mandated coverage in their base level of benefits, specific rates must be developed so that contract holders who select such optional coverage can be appropriately charged for them.

Because companies do not ordinarily develop separate rates for most benefits, much of the premium data reported to the Commission has been developed for the express purpose of complying with § 38.2–3419.1.^11^ The percent of overall average premium attributable to each mandated benefit is computed by dividing the estimated premium applicable to each mandated benefit by the overall average premium for all contracts subject to the reporting requirement.

Estimated premium impact is applied to an individual or family “Standard Policy” for a 30-year old male in the Richmond, VA area with a policy in the standard premium class including $250 deductible, $1,000 stop-loss limit, 80 % co-insurance factor and a $250,000 policy maximum.

## Discussion

### Insured population

The actual numbers of companies filing reports showed a steady decline over this period, dropping substantially after 2010 (Table 2), an indication of the healthcare industry consolidation starting after passage of the Patient Protection and Affordable Care Act of 2010.^12^ The decrease in number of coverage units may partly reflect the decrease in private health insurance coverage in Virginia between 2008 and 2012 documented in a Census Bureau report in 2013.^13^

### Claim costs

Average claim costs for lymphedema treatment exhibited a 4–9 % annual growth over the initial 10 years of the Virginia mandate for both individual and group policies (Table 4). The growth trends, however, displayed different characteristics (Fig. 1). Individual contract claim costs displayed an initial growth the first 3 years, after which it remained virtually constant at around $1.75 per contract per year. Group contract claims, however displayed an unstable growth over the 10-year period averaging 9.18 % per year, virtually doubling during that period. Neither the office visits nor the hospitalizations (Fig. 3) support the idea that increased utilization was responsible for the rising costs. That rising lymphedema treatment costs rose due to inflation is implied by the fact that while the dollar amount of lymphedema claims is rising, the percent of average contract claims remains essentially constant, ie the cost of healthcare is rising and it is dragging lymphedema costs along. The magnitude of the growth matches the eight percent per year growth in healthcare costs over the same period. The unevenness may be a reflection of the post-PPACA turmoil in the group business. Insurers should note however the beneficial effects of lymphedema treatment in reduced utilization of physician’s and therapist’s services for lymphedema and a reduction in hospital stays for lymphedema or cellulitis treatment.

**Table 4:** 10-year claims statistics- insurance plans, & HMO policies

Cost per contract	mean	STDDEV	Slope %/Y
Individual contract	$1.59	$0.24	3.95 %
Group contract	$3.24	$0.98	9.18 %
Percent total claims	mean	STDDEV	Slope % /Y
Individual policies	0.053	0.008	−0.57 %
Group policies	0.089	0.015	4.70 %
HMO individual	0.012	0.011	14.14 %
HMO group	0.033	0.015	5.69 %

Statistical analysis of abstracted data using Microsoft® Excel® 2008 for Mac, Version 12.3.6 installed on Apple iMac under OSX Version 10.9.5 operating system. Mathematical functions AVERAGE, STDEVP AND SLOPE were utilized to determine the means, standard deviations, and slopes of the data, respectively

### Lymphedema claims as a percent of total contract claims

Lymphedema claims constituted 0.053 % ± 0.008 % standard deviation (SD) of total claims for individual contracts (Table 4). For group policies they constituted 0.073 % for the first 4 years, and then rose to 0.100 % for the last 6 years (Fig. 2). Lymphedema claims constituted 0.012 % ± 0.011 % SD and 0.033 % ± 0.015 % SD of total HMO claims for individual and group contracts respectively. The only trend observable is that HMO lymphedema claims as a percentage of total claims have been monotonically rising since 2010 (Fig. 2).

#### The salient conclusion is that lymphedema treatment costs are less than one thousandth of the total claims costs in all types of insurance contracts

##### Estimated premium impact of a lymphedema treatment mandate

**Fig. 4:**
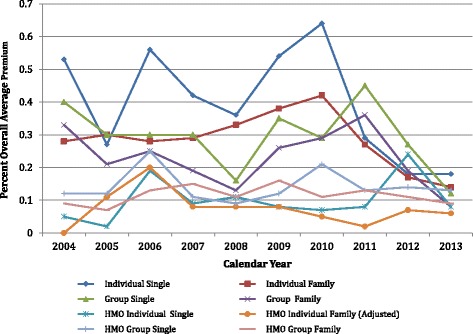
Estimated Premium Impact of Lymphedema Mandate. Source: Data abstracted from Tables 1, 2, 3, 4, and 17 of Private, Group, HMO Contract reports

The estimated premium impact of the lymphedema mandate ranged 0.14–0.64 % of the overall average contract premium on individual contracts, and 0.00 to 0.45 % on HMO contracts (Fig. 4). Estimated premiums are an underwriter’s best guess as to what future premiums must be to adequately cover estimated future claims, based on prior years’ claims. A detailed inspection of the statistics of claim data and estimated premiums show that the estimated premium impacts vary wildly between 4 and 10 times the claims impact for the first 8 years while sufficient claims experience was being gathered. But in the last 2 years estimated premium impact for all types of policies converged to approximately two times projected claims at 0.06 to 0.18 % of overall average premiums indicating a maturing of the actuarial projections of lymphedema impact on premiums. It is this convergence based on 10 years of claims experience that is evident in this graphic.

#### After 10 years of actuarial experience with the lymphedema mandate the premium impact for all types of contracts converged to less than 0.2 % of the contract total premiums

##### Utilization

Starting in 2008, the fifth year of the mandate, the number of visits to physicians and therapists dropped from 0.1 to 0.06 visits per year per contract. This was accompanied by a trend towards 0 days of hospitalization for lymphedema (Fig. **3**). Reductions in office visits and hospitalization days over the full 10-years was 7.32 and 6.06 % per year. Similar reductions have been noted in the literature. Prof. E. Földi concluded [2] from a trial she describes in 1996 “… in women with arm lymphedema after treatment of breast cancer, recurrent DLA (Dermatolymphangioadenitis or cellulitis) attacks can nearly be eliminated by improvements in arm swelling by CPT (combined physiotherapy) (phase I)”

In a 1998 trial involving 299 patients with upper and lower limb lymphedema, Ko, Lerner, Klose et al. [5] observed that the incidence of infections decreased from 1.10 infections per patient per year to 0.65 infections per patient per year after a complete course of CDP (combined decongestive physiotherapy).

More recent studies [3] and [6] involving retrievals from a large claims database involved cancer survivors with diagnosed lymphedema who had obtained a pneumatic compression device (PCD) as part of the treatment of their lymphedema.

Brayton et al. [3] compared health outcomes and costs in the year before and the year after receipt of the PCD. PCD use was associated with a decrease in hospitalizations (45 % to 32 %, *p* < 0.0001), outpatient hospital visits (95 to 90 %, *p* < 0.0001), cellulitis diagnoses (28 to 22 %, *p* = 0.003), and physical therapy use (50 to 41 %, *p* < 0.0001).

Karaca-Mandic et al. [6] showed that use of an advanced PCD (APCD) was associated with similar reductions in adjusted rates of cellulitis episodes from 21.1 to 4.5 % in the cancer-related lymphedema cohort and 28.8 to 7.3 %in the non cancer-related lymphedema cohort (*P* < .001 for both). Lymphedema-related manual therapy was reduced from 35.6 to 24.9 % in the cancer cohort and from 32.3 to 21.2 % in the non cancer cohort (*P* < .001 for both). And outpatient visits were reduced from 58.6 to 41.4 % in the cancer cohort and from 52.6 to 31.4 % in the non cancer cohort (*P* < .001 for both).

We believe that these are notable trends, supported by ten years of data from Virginia, that verify the basic tenet that treatment of lymphedema reduces the incidence of infections requiring medical attention and hospitalization.

## Virginia, Massachusetts, California and North Carolina mandate reports

### Preliminary mandate commission analyses

A description of the mandate commission analyses of the impacts of lymphedema treatment mandates in Virginia was published by Stout NL, Weiss R, Feldman JL, et al. as Table 3 of Ref. 4. Some of the conclusions relating to the fiscal impact of a lymphedema treatment mandates in Virginia, Massachusetts, California and North Carolina will be covered below. A common incorrect assumption in all these analyses is that the pre-mandate insurance coverage includes coverage of lymphedema treatment to current medical standards. This claim can be shown to be false by inspecting the medical policies of health insurers in these states prior to institution of a lymphedema treatment mandate, many of which did not cover compression bandage systems, compression garments and devices, had therapy limitations based on arbitrary limits instead of medical need, and did not provide sequential pneumatic compression devices specifically designed to treat lymphedema.

### Virginia pre-mandate analysis

Respondents to the Commonwealth of Virginia (COVA) Bureau of Insurance survey in 2002^7^, on the estimated cost impact of a lymphedema mandate, provided cost figures that ranged from less than $0.15 to $2.00 per month ($1.80 to $24.00 per year) per standard individual policy holder, and from $0.02 to $5.53 per month ($0.24 to $66.36 per year) per standard group certificate, to provide the coverage required by Virginia House Bill 383/1737. Insurers providing coverage on an optional basis provided cost figures of $0.25 to $5.58 per month ($3.00 to $66.96 per year) per individual policyholder and from $0.25 to $3.98 per month ($3.00 to $47.76 per year) per group certificate holder for the coverage required by House Bill 383/1737.^2^ Only the lowest of the provider projections in 2002 have turned out to have any validity.

### California mandate analysis

In 2005 the California Health Benefits Review Program (CHBRP)^5^ estimated an increase of 0.0003 % or $0.01 per person per year for implementing California Assembly Bill AB-213 Liu, Health Care Coverage for Lymphedema. The basic assumption in the financial analysis was that California insurers already cover the treatment of lymphedema, including manual lymph drainage and compression garments (with some limitations). Their estimate of lymphedema prevalence among patients under 65 years of age was 0.07 % based on data from the Milliman claims database using ICD-9-CM diagnostic codes of 457.0, 457.1 and 457.2 (the last code not being a diagnosis of lymphedema, and omitting code 757.0 lower limb congenital lymphedema). Increased utilization of lymphedema services was assumed to be between 1.48 and 2.00 % due to increases in DME, compression garments, and therapy visits due to increased awareness of the availability of coverage. Utilization of pharmaceuticals, physician visits, hospitalization and all other medical services were assumed to remain the same as baseline. None of these assumptions are borne out with the Virginia data.

### Massachusetts mandate analysis

A lymphedema treatment cost survey was performed in 2010 as part of a Massachusetts lymphedema mandate study.^6^ This analysis, like the earlier California analysis, assumed that the treatment of lymphedema was covered prior to introduction of a new treatment mandate. Reimbursements for lymphedema procedures and devices, as derived by a retrospective survey of 2008 claims based on lymphedema ICD-9-CM and CPT codes, averaged $0.144 per member per year for fully insured contracts and $0.324 per patient per year for self-insured contracts.

Patients with lymphedema use a wide range of services. Overall utilization among lymphedema patients is low, with treatment considerably underutilized. Claims data show, for example, that around 12 % of lymphedema patients utilize physical or occupational therapy, around 20 % use compression garments, and fewer than 10 % use manual lymphatic drainage.

The 2010 analysis of Massachusetts Senate Bill 896, a lymphedema mandate bill, estimated per member per month incremental costs of a lymphedema mandate (Table 5 of the Massachusetts mandate report^6^) of $0.006, $0.028 and $0.073 (equivalent to $0.072, $0.336 and $0.876 per member per year) for low, middle and high scenarios for coverage of lymphedema treatment in the year 2011. Scenarios varied the distribution of severities of lymphedema and therefore the expense of treating. The different scenarios also included postulated plans with no treatment limits, and plans with annual limits on physical therapy and caps on lymphedema durable medical equipment, prosthetics, orthotics and supplies (DMEPOS), eg devices, bandages, compression garments.

#### These estimates are of the same magnitude as the Virginia actuals

They are somewhat lower as they represent the incremental cost of a mandate for services partially already provided in baseline coverage, while the Virginia data represents total collected cost of lymphedema treatment.

### North Carolina Fiscal Impact of a lymphedema mandate

Aon Consulting, the consulting actuary for the North Carolina State Health Plan for Teachers and State Employees, and Hartman & Associates, the consulting actuary for the North Carolina General Assembly's Fiscal Research Division, estimated that a lymphedema mandate would have no financial impact on the employees’ plan given that coverage for lymphedema-related treatment is already a covered benefit.^9^

### Incremental VS total costs of a lymphedema treatment mandate

It is important to make a distinction between the calculated impacts of a lymphedema mandate on premiums and the total cost of lymphedema treatment.

In a 2002 Virginia State Corporation Commission’s Bureau of Insurance survey of the top 60 writers of accident and sickness insurance in Virginia, 36 companies then writing applicable business in Virginia responded. Of the 36, 26 companies (72 %) claimed to already provide the coverage required by Virginia House Bill 383/1737 that was later implemented.^7^

A similar observation was made in the Massachusetts mandate review.^6^ “All plans provide coverage for treatment for active lymphedema, but many, if not most, policies have limitations on the number of therapy visits (20–24 per year) and limits on reimbursements for supplies and devices such as compression garments and pneumatic compressors and related appliances. In particular some of the garments are regarded, according to responses to the Division’s survey, as durable medical equipment (DME) and subject to policy DME limits.”

The North Carolina Fiscal Research Division simply stated “Note that the calculation of an estimated premium impact based on collected lymphedema treatment costs in Virginia does not represent additional cost due to the mandate, since the majority of providers already provided some treatment of lymphedema prior to the mandate.^9^

These so-called additional costs of a mandate are to be contrasted with the segregated costs of lymphedema treatment in Virginia, which reflect all costs of lymphedema treatment whether they were provided before or after the mandate went into effect.

### Benefits of lymphedema treatment

None of these analyses accounted for significant avoided costs due to reduced infection that could be passed on to the customer as reduced premiums. The California analysis^5^ author notes “Costs may be easier to identify than the long-term benefits of this legislation, and so the absence of information regarding benefits in this section should not be an indication of the benefits of this legislation.”

A custom retrieval from the 2003 California Patient Discharge Database was made by Weiss in 2007 [7] to determine the projected savings by management of lymphedema, through the reduction of lymphedema-related cellulitis. A saving of approximately $2.54 per year per insured Californian was calculated in this pilot retrieval, which exceeds the reported 2004 costs of $1.12–$2.17 from the Virginia data, implying a positive return on the investment for early lymphedema treatment.

### Completeness of reported mandate costs

According to the Virginia Code the covered benefits for lymphedema treatment include “equipment, supplies, complex decongestive therapy, and outpatient self-management training and education”^2^. Costs are reported by providers and insurers to the State of Virginia using Form MB-1.^11^ The instructions are not explicit as to what costs are to be included except to request “specific claim data” for each mandated benefit. Specific claim codes are required to be collected include CPTs 97124, 97140 and 97535^10^ covering massage therapy, manual lymph drainage, and outpatient self-management training and education. It is not clear, however, whether collected costs also include costs of physician and therapist evaluation or costs of compression bandages, garments, devices and supplies used in the daily management of lymphedema, or CPT Code 97016 Vaso-pneumatic device (lymphedema pump) therapy, sometimes used for the treatment of lymphedema. It is not clear whether charges for this procedure are collected by diagnostic code and whether they are collected by ICD-9-CM code for lymphedema per the Form MB-1 instructions.

Actuarial methods for determination of estimated premium impact for these reports appear to be somewhat inconsistent. For example, whereas the percent of total contract claims for individual policies is relatively level at 0.04–0.06 % over the 10-year study period, the estimated premium impact ranges between 0.14 and 0.64 % for the same policies. Reporting instructions^11^ require “for the purpose of this report it is required that a dollar amount [of the annual premium for each policy] be assigned to each benefit and provider based on the company's actual claim experience, …”. In the individual health insurance market, the percent of premiums used to pay claims typically ranges from about 70 to 85 %.^14^ It is therefore hard to reconcile the estimates of premium impact that is 3–10 times the claim costs attributable to lymphedema treatment. For this reason the collected actual claim costs and utilization data are more to be trusted than the estimated premium impact.

## Conclusions

An estimate of the cost of lymphedema treatment from an insurer’s viewpoint was made using the actual claims data in Virginia, where a lymphedema treatment mandate has been in effect since January 1, 2004. It is an upper bound since reduction in total claim costs due to resulting lower cellulitis rates are not used to reduce estimated premiums.

The average actual lymphedema claim cost was $1.59 per individual contract and $3.24 per group contract for the years reported, representing 0.053 and 0.089 % of average total claims. These actual costs are compared with pre-mandate state mandate commission estimates for proposed lymphedema mandates from Virginia, Massachusetts and California. Pre-mandate cost estimates tend to overestimate the actual costs of lymphedema treatment.

The Virginia data confirmed previous clinical data that the treatment of lymphedema by management of swelling results in lower medical costs and fewer hospitalizations. This is a potent model for reduction in healthcare costs while improving the quality of care for cancer survivors and others suffering with this chronic progressive condition.

## Footnotes

1 United States Senate Committee on Finance, Chronic Care Workgroup letter to stakeholders, dated May 22, 2015; Available from http://www.finance.senate.gov/newsroom/chairman/release/?id=9f9f2d3e-401e-409b-a53a-22bbe3f56f2c. Accessed on September 30, 2015.

2 Virginia House Bill No. 1737, “A bill … relating to health insurance coverage for lymphedema” by Delegate Leo C. Wardrup, Jr. Offered January 8, 2003. Available from http://lis.virginia.gov/cgi-bin/legp604.exe?031+ful+HB1737ER. Accessed on September 30, 2015.

3 Code of Virginia § 38.2-3419.1. Report of costs and utilization of mandated benefits. Available from http://leg1.state.va.us/cgi-bin/legp504.exe?000+cod+38.2-3419.1. Accessed on September 30, 2015.

4 Virginia, State Corporation Commission Series. Report of the State Corporation Commission to the Governor and the General Assembly of Virginia: The Financial Impact of Mandated Health Insurance Benefits and Providers Pursuant to Section 38.2-3419.1 of the Code of Virginia: [year] Reporting Period. Reports are listed for the reporting years covered in Table 1, and are available at the Virginia Department of Insurance website at URL: http://leg2.state.va.us/DLS/h&sdocs.nsf/Search+All/?SearchView&SearchOrder=4&query=38.2-3419.1. Accessed October 4, 2016.

5 California Health Benefits Review Program. Analysis of Assembly Bill 213 Health Care Coverage for Lymphedema - A Report to the 2005-2006 California Legislature. April 7, 2005. Available at http://chbrp.org/documents/ab_213final.pdf. Accessed on September 30, 2015.

6 Massachusetts Division of Health Care Finance and Policy. Review and Evaluation of Proposed Legislation Entitled: An Act Relative to Women’s Health and Cancer Recovery, Senate Bill 896 - Provided for The Joint Committee on Public Health. December 2010. Appendix: Compass Health Analytics, Inc. Actuarial Assessment of Senate Bill 896: An Act Relative to Women’s Health and Cancer Recovery – Prepared for Commonwealth of Massachusetts Division of Health Care Finance and Policy, June 18, 2010. Available at http://archives.lib.state.ma.us/bitstream/handle/2452/101824/ocn711076017.pdf?sequence=1&isAllowed=y. Accessed on September 30, 2015.

7 Virginia Special Advisory Commission on Mandated Health Insurance Benefits. Report of the Special Advisory Commission on Mandated Health Insurance Benefits, House Bill 383, Mandated Coverage of Lymphedema, Commonwealth of Virginia, Richmond, 2003. Available at
http://leg2.state.va.us/DLS/H&SDocs.NSF/Published%20by%20Year? Accessed on September 30, 2015 2003-RD15 Mandated Coverage of Lymphedema.

8 Individual health plans are contracts between an individual and the insurance provider (insurance company or HMO), and can cover an individual or the individual and spouse and dependents. Group health plans are offered by employers as part of an employee benefits package. Both individual and group plans are considered Private health insurance for Census Department Purposes. State health plans are offered to Commonwealth of Virginia (COVA) employees, their families, and retirees, as well as Medicaid participants, and include group plans and HMO plans from a provider selected by the State. In all these cases the plans must provide lymphedema treatment coverage per State Law. Not included in these statistics are Medicare plans, which are not subject to Virginia State law.

9 North Carolina Fiscal Research Division. General Assembly of North Carolina, Session 2009, Legislative Actuarial Note, Health Benefits, Bill Number: House Bill 535. April 17, 2009 http://www.ncleg.net/Sessions/2009/FiscalNotes/House/PDF/HAH0535v1n1.pdf. Accessed on September 30, 2015.

10 Virginia. CPT and ICD-9-CM Codes to be used in filling out Form MD-2 for reporting costs of mandated benefits (2011). http://www.scc.virginia.gov/boi/co/health/ltcmed/cpt_codes.pdf. Accessed on September 30, 2015.

11 Virginia Administrative Code 14VAC5-190-80:1 APPENDIX A. FORM MB-1 INSTRUCTIONS AND INFORMATION. http://leg1.state.va.us/cgi-bin/legp504.exe?000+reg+14VAC5-190-80Z1. Accessed on September 30, 2015.

12 Haislmaier EF. Health Care Consolidation and Competition after PPACA – Testimony before Committee on the Judiciary Subcommittee on Intellectual Property, Competition and the Internet, United States House of Representatives, May 18, 2012. Available at http://www.heritage.org/research/testimony/2013/06/health-care-consolidation-and-competition-after-ppaca. Accessed on October 4, 2016.

13 U.S. Department of Commerce Economics and Statistics Administration U.S. CENSUS BUREAU, Mitigating the Loss of Private Insurance With Public Coverage for the Under-65 Population: 2008 to 2012, Jonathan Rodean, September 2013. http://www.census.gov/content/dam/Census/library/publications/2013/acs/acsbr12-11.pdf. Accessed on August 5, 2016.

14 American Academy of Actuaries. Critical Issues in Health Reform: Premium Setting in the Individual Market, March 2010. Available at http://actuary.org/files/publications/premiums_settings_mar2010.pdf. Accessed 26 Aug 2016.
