# Erratum to: Cost of a lymphedema treatment mandate-10 years of experience in the Commonwealth of Virginia

**DOI:** 10.1186/s13561-016-0125-3

**Published:** 2016-10-19

**Authors:** Robert Weiss

**Affiliations:** 10671 Baton Rouge Avenue, Porter Ranch, CA 91326 USA

## Erratum

Unfortunately both the HTML and the PDF versions of this article [1] on the SpringerOpen web site contained typographical errors in the superscripted citations to the list of footnotes/endnotes and in some hyperlinks to tables. Two URLs to the data reports have been expanded to take the reader directly to the full set of mandated annual reports without further searching.

**Changes to in-text citations in HTML Version**

**Table:** 

**Section or Subsection Title**	**Containing Text**	**Corrected Text**
CPT and ICD-9-CM codes collected	…reporting rules^2,,3^ require …	…reporting rules^2,3^ require …
Insured population	… Care Act of 2010.	… Care Act of 2010^12^.
Insured population	…Bureau report in 2013.	…Bureau report in 2013^13^.
Virginia pre-mandate analysis	Virginia pre-mandate analysis	Virginia pre-mandate analysis^6^
California mandate analysis	California mandate analysis	California mandate analysis^5^
Massachusetts mandate analysis	Massachusetts mandate analysis	Massachusetts mandate analysis^7^
North Carolina …mandate	North Carolina …mandate	North Carolina …mandate^8^
Benefits of lymphedema treatment	The California analysis^9^ author..	The California analysis^5^ author..
Completeness of reported mandate …	Reporting instructions^12^ require	Reporting instructions^3^ require
Completeness of reported mandate …	…from about 70 to 85 %^13^.	…from about 70 to 85 %^14^.
Completeness of reported mandate …	… to lymphedema treatment^14^.	… to lymphedema treatment.

**Deletion of hyperlinks in HTML version to tables in other documents**

**Table:** 

**Section or Subsection Title**	**Containing Text**	**Corrected Text**
Population Coverage	… on Table 3 of reference [4].	… on Table 3 of reference [4].
Preliminary mandate commission …	… as Table 3 of Ref. 4.	… as Table 3 of Ref. [4].

**Correction of URLs in HTML version**

In Table 1, URL should be: http://leg2.state.va.us/DLS/h&sdocs.nsf/Search+All/?SearchView&SearchOrder=4&query=38.2-3419.1

In Footnote 4, URL should be: http://leg2.state.va.us/DLS/h&sdocs.nsf/Search+All/?SearchView&earchOrder=4&query=38.2-3419.1

**Changes to in-text citations in PDF Version**

**Table:** 

Section or Subsection Title	Containing Text	Corrected Text
Annual data reports	…in place a statute that requires	…in place a statute^3^ that requires
Annual reports to the VA governor	…insurance, and HMOs,^3^ …	…insurance, and HMOs,^4^ …
Benefits of lymphedema treatment	The California analysis^4^ author..	The California analysis^5^ author..
Completeness of reported mandate …	Reporting instructions^11^ require	Reporting instructions^10^ require
Completeness of reported mandate …	…from about 70 to 85 %^12^.	…from about 70 to 85 %^14^.
Completeness of reported mandate …	… to lymphedema treatment^14^.	… to lymphedema treatment.

**Correction of URLs in PDF version**

In Table 1, URL should be: http://leg2.state.va.us/DLS/h&sdocs.nsf/Search+All/?SearchView&SearchOrder=4&query=38.2-3419.1

In Endnote 2, URL should be: http://leg2.state.va.us/DLS/h&sdocs.nsf/Search+All/?SearchView&SearchOrder=4&query=38.2-3419.1

In Endnote 11, URL should be: http://www.heritage.org/research/testimony/2013/06/health-care-consolidation-and-competition-after-ppaca. Accessed on September 13, 2016.
